# The Swedish version of the TeamSTEPPS® teamwork attitudes questionnaire (T-TAQ): A validation study

**DOI:** 10.1186/s12913-021-06111-1

**Published:** 2021-01-30

**Authors:** Marie Louise Hall-Lord, Carina Bååth, Randi Ballangrud, Anna Nordin

**Affiliations:** 1 Department of Health Science, Faculty of Medicine and Health Sciences, Norwegian University of Science and Technology, Gjøvik, Norway; 2 Department of Health Sciences, Faculty of Health, Science and Technology, Karlstad University, Karlstad, Sweden; 3 Faculty of Health and Welfare, Østfold University College, Fredrikstad, Norway

**Keywords:** Attitude, Interprofessional, Questionnaire, Team training, Teamwork, Validation studies

## Abstract

**Background:**

Effective teamwork is essential for delivering safe health care. It is important to increase patient safety in healthcare by conducting interprofessional team training with both healthcare professionals and undergraduate students. Validated questionnaires that evaluate team training activities contribute to valuable knowledge regarding changes in attitudes toward teamwork. The aim of the study was to test the reliability and structural validity of the Swedish version of the TeamSTEPPS® Teamwork Attitudes Questionnaire (T-TAQ).

**Methods:**

The study had a cross-sectional design. Four hospitals in three health care regions in Sweden participated in the study. In total, 458 healthcare professionals, response rate 39.4%, completed the questionnaire. The T-TAQ, which consists of 30 items and covers five dimensions (Team Structure, Leadership, Situation Monitoring, Mutual Support and Communication), was translated to Swedish. A paper version of the T-TAQ was distributed to healthcare professionals (physicians, registered nurses, midwives, nursing assistants and allied health professionals) from the hospitals. Reliability and validity were tested using Cronbach’s alpha and confirmatory factor analysis.

**Results:**

Cronbach’s alpha was 0.70 for the total T-TAQ and ranged from 0.41 to 0.87 for the individual dimensions. The goodness-of-fit indexes in the confirmatory factor analysis (Model 2) revealed a normed chi-square of 2.96, a root mean square error of approximation of 0.068, a Tucker-Lewis index of 0.785 and a comparative fit index of 0.808.

**Conclusions:**

The Swedish version of the T-TAQ has some potential to measure healthcare professionals’ general attitudes toward the core components of teamwork in hospital settings. Further validation studies of the Swedish version of the T-TAQ are required, with samples representing both healthcare professionals and students from various healthcare disciplines and educational levels.

## Background

There is an increasing focus on effective interprofessional teamwork to improve communication and collaboration among healthcare professionals to enhance patient safety in healthcare [1]. Failures in communication [2–5] and a lack of coordination [6] between healthcare professionals may lead to adverse events in patients [4]. Although interventions in healthcare should include organizational factors to have a significant impact on patient safety and quality of care [7], team training for healthcare professionals and undergraduate students in various healthcare disciplines is important [8]. Evaluating the impact of training activities with validated measurements is vital, and change in attitudes can be used as a measurement of learning outcome [9] and contribute to valuable knowledge regarding changes in attitudes toward teamwork.

Healthcare provided by teams rather than individuals requires collaboration between healthcare personnel from various professions. Potential solutions to building better health care teams and teamwork that is more effective can involve educational interventions. Salas et al. [10] divided effective teamwork into the “Big five” dimensions (team leadership, mutual performance monitoring, backup behavior, adaptability, and team orientation), which are coordinated by the underlying mechanisms mutual trust, closed-loop communication and shared mental models. To achieve the five dimensions of effective teamwork, team members must respect and trust each other, have good communication skills and have a shared mental model to facilitate the team’s progression toward goal attainment that promotes common understanding and action. The relationship between communication and patient safety [11, 12] indicates the need to educate healthcare professionals and healthcare students in interprofessional team training [13, 14],

To improve quality and safety in healthcare in the US, the Department of Defense and the Agency for Healthcare Research and Quality (AHRQ) developed the Team Strategies and Tools to Enhance Performance and Patient Safety (TeamSTEPPS®) program, which is an evidence-based program based on the theory of the “Big five” dimensions of effective teamwork developed by Salas et al. [10]. TeamSTEPPS® includes Team Structure in addition to four important teamwork competences: Leadership, Situation Monitoring, Mutual Support and Communication. The program provides knowledge of the TeamSTEPPS® principles and on how to effectively work in interprofessional teams. TeamSTEPPS® incorporates simulation as a training tool, which is common when educating healthcare professionals and undergraduate students in interprofessional teamwork [15]. Interprofessional team training with TeamSTEPPS® and simulation shows improved communication, team performance, patient outcomes [15] and patient safety culture [16–18].

The American Institute of Research developed the TeamSTEPPS® Teamwork Attitudes Questionnaire (T-TAQ) to measure individual attitudes related to the core components of teamwork [19]. The T-TAQ has been validated by Baker et al. [20] and can be used to understand the effects of team training, as it is administrated before and after the training. The questionnaire has been used in several studies evaluating team training with undergraduate students from various disciplines [21–23], with nursing students [24, 25] and with individuals continuing interprofessional education [18, 26, 27]. There are few tools based on a theoretical framework measuring teamwork attitudes available in Sweden and we judged the T-TAQ to fit in the context of a Swedish health care system, since the concepts and content used in the questionnaire are in agreement with Swedish terminology. The T-TAQ was translated with the permission of the United States AHRQ from the TeamSTEPPS® 2.0 National Implementation (accessible at http://teamstepps.ahrq.gov/). New validation efforts are required to determine whether a questionnaire translated into another language and used in another culture is a valid measure of the construct [28]. The aim of this study was to test the reliability and structural validity of the Swedish version of the TeamSTEPPS® Teamwork Attitudes Questionnaire (T-TAQ).

## Methods

### The questionnaire and the translation process

The T-TAQ consists of 30 items and covers five dimensions: Team Structure, Leadership, Situation Monitoring, Mutual Support and Communication. Each dimension has six items with five response options ranging from 1 = strongly disagree to 5 = strongly agree on a Likert scale. Four items are negatively worded, including three items in the Mutual Support dimension and one item in the Communication dimension. The scores for the total scale and for each dimension were calculated by adding all items and dividing the score by the number of items in each dimension and in the total scale [19, 20].

Brislin [29] inspired the translation of the T-TAQ. A Swedish professional bilingual translator translated the original English version of the questionnaire from English to Swedish. We reviewed the translated version of the T-TAQ with some minor semantic and conceptual adjustments. Another professional bilingual translator performed back-translation of the reviewed Swedish version to English. Then, the research group assessed the original version and the back-translated version of the T-TAQ with only some minor revisions in the translated questionnaire. A pilot test of the Swedish T-TAQ was carried out with 15 healthcare professionals (three physicians, eight registered nurses, two midwives, one nursing assistant and one physiotherapist) working in clinical practice. The participants were asked to comment if the items were understandable and clear. Due to their replies, some further language changes were made in the questionnaire.

### Design

This study had a cross-sectional design.

### Settings and sample

Four hospitals (hospital A, hospital B, hospital C, hospital D) in three health care regions in Sweden were included. In hospital A, the operating room and the medical-, gynecological-, obstetric-, and intensive care wards participated. In hospital B, the emergency room and medical care wards participated. In hospital C and hospital D, the obstetric care wards participated. All frontline healthcare professionals in the wards, including physicians, registered nurses, midwives, nursing assistants, and allied health professionals (*N* = 1176), were invited to participate in the study.

### Data collection

The chief manager in the ward and members of the research group informed the healthcare professionals about the study. Between September and December 2018, the healthcare professionals obtained a paper version of the T-TAQ together with a letter containing information about the study. Completed questionnaires were returned to the research group anonymously in self-addressed envelopes.

### Statistics

Data analysis was conducted using IBM SPSS Statistics for Windows, version 25.0 (Armonk, New York) and IBM AMOS version 25.0. The four negatively worded items in the questionnaire were reverse-coded and a mean score was calculated for the total scale and for each teamwork dimension. Internal consistency (measured by Cronbach alpha) was tested for the total questionnaire and each teamwork dimension. Corrected item-total correlation and Cronbach alpha whether an item was deleted were calculated for the five individual dimensions.

Participants with no missing data were included in the confirmatory factor analysis (CFA). Structural validity by CFA tested the a priori hypothesis based on the theoretical understanding of the structure of factors, in this case, by analyzing the empirical data of the five dimensions of T-TAQ. CFA yields goodness-of-fit indexes, which illustrate how well the hypothesized model matches the data [28]. First, the goodness-of-fit indexes evaluated how well the data corresponded to the hypothesized five-factor model (Model 1). Second, since the four reverse-coded items indicated low corrected item-total correlation and low factor loadings, we decided to build a model with a possible better fit to the data (Model 2). In this post-hoc modification, the error variances for the four negatively worded items were correlated. Common goodness-of-fit indexes were used to assess the strength of the two models [28, 30], including the chi-square goodness of fit (x^2^), normed chi-square (chi-square/df), root mean square error of approximation (RMSEA), Tucker-Lewis index (TLI) and comparative fit index (CFI). RMSEA is one of the most informative criteria in CFA and takes into account the error of the approximation in the population [30]. TLI and CFI evaluate the goodness of fit of a hypothesized model and compare this model with a null model [28, 30]. There are criteria for the goodness-of-fit indexes for determining model fit. The chi-square goodness of fit (x^2^) should have a *p* value of > 0.05 and the normed chi-square value should be < 3. A RMSEA value less than 0.06 indicate good fit and values as high as 0.08 represent errors of approximation in the population [30]. For TLI and CFI values close to 0.95 indicate a good fit [31].

## Results

In total, 458 of the healthcare professionals (response rate 39.4%) completed the T-TAQ (Table 1).

**Table 1:** The healthcare professionals, invited and responded

	Invited	Responded	Responded per profession group
	N	N (%)	%
All healthcare professionals	1176	458 (39.4)	
Profession			
Physician	229	68 (14.9)	29.7
Registered nurse	387	136 (29.7)	35.1
Midwife	222	120 (26.2)	54.1
Nursing assistant	313	111 (24.2)	35.5
Allied health professional	25	7 (1.5)	28.0
Missing		16 (3.5)^a^	

^a^Did not respond to the item of profession

Table 2 shows the corrected item-total correlation and Cronbach’s alpha for each dimension and whether the item was deleted.

**Table 2:** Summary of reliability for T-TAQ items and dimensions (*N* = 458)

	Corrected item-total correlation	Cronbach alpha - item deleted	Cronbach alpha
**Team Structure**			0.70
1. It is important to ask patients and their families for feedback regarding patient care.	0.45	0.66	
2. Patients are a critical component of the care team.	0.36	0.68	
3. This facility’s administration influences the success of direct care teams.	0.38	0.68	
4. A team’s mission is of greater value than the goals of individual team members.	0.48	0.64	
5. Effective team members can anticipate the needs of other team members.	0.52	0.64	
6. High-performing teams in health care share common characteristics with high-performing teams in other industries.	0.43	0.66	
**Leadership**			0.87
7. It is important for leaders to share information with team members.	0.70	0.85	
8. Leaders should create informal opportunities for team members to share information.	0.71	0.85	
9. Effective leaders view honest mistakes as meaningful learning opportunities.	0.67	0.85	
10. It is a leader’s responsibility to model appropriate team behavior.	0.66	0.86	
11. It is important for leaders to take time to discuss with their team members plans for each patient.	0.68	0.85	
12. Team leaders should ensure that team members help each other out when necessary.	0.66	0.86	
**Situational Monitoring**			0.74
13. Individuals can be taught how to scan the environment for important situational cues.	0.39	0.72	
14. Monitoring patients provides an important contribution to effective team performance.	0.42	0.72	
15. Even individuals who are not part of the direct care team should be encouraged to scan for and report changes in patient status.	0.47	0.71	
16. It is important to monitor the emotional and physical status of other team members.	0.55	0.69	
17. It is appropriate for one team member to offer assistance to another who may be too tired or stressed to perform a task.	0.53	0.69	
18. Team members who monitor their emotional and physical status on the job are more effective.	0.57	0.67	
**Mutual Support**			0.41
19. To be effective, team members should understand the work of their fellow team members.	0.23	0.37	
20. Asking for assistance from a team member is a sign that an individual does not know how to do his/her job effectively.^a^	0.18	0.40	
21. Providing assistance to team members is a sign that an individual does not have enough work to do.^a^	0.40	0.26	
22. Offering to help a fellow team member with his/her individual work tasks is an effective tool for improving team performance.	0.07	0.45	
23. It is appropriate to continue to assert a patient safety concern until you are certain that it has been heard.	0.22	0.37	
24. Personal conflicts between team members do not affect patient safety.^a^	0.20	0.38	
**Communication**			0.63
25. Teams that do not communicate effectively significantly increase their risk of committing errors.	0.47	0.55	
26. Poor communication is the most common cause of reported error.	0.53	0.52	
27. Adverse events may be reduced by maintaining an information exchange with patients and their families.	0.50	0.54	
28. I prefer to work with team members who ask questions about information I provide.	0.37	0.59	
29. It is important to have a standardized method for sharing information when handing off patients.	0.38	0.59	
30. It is nearly impossible to train individuals how to be better communicators^a^.	0.02	0.72	

^a^Reverse-coded item

The corrected item-total correlations were above 0.30 for items in three (Team Structure, Leadership, Situational Monitoring) of the five dimensions. The corrected item-total correlations for five out of six items in the Mutual Support dimension and one item in the Communication dimension were below 0.30. Cronbach’s alpha was 0.70 for the total T-TAQ and ranged from 0.41 (Mutual Support) to 0.87 (Leadership) for the five dimensions. Cronbach’s alpha showed a higher score in the Mutual Support dimension (0.45) and in the Communication dimension (0.72) whether an item in each dimension was deleted.

Participants with no missing data were included in the CFA (*N* = 423). The first model showed values below the recommended limits of the CFA criteria indexes (see Table 3).

**Table 3:** Confirmatory factor analysis fit indexes (*N* = 423)

	CFA index criteria	Model 1 (Unmodified)	Model 2 (Modified)
Chi-square (df), *p*-value	*p* > 0.05	1356 (395), *p* < 0.001	1152 (389), *p* < 0.001
Normed chi-square	< 3	3.43	2.96
RMSEA (CI)	< 0.8	0.076 (0.072, 0.080)	0.068 (0.064, 0.073)
TLI	> 0.90	0.734	0.785
CFI	> 0.90	0.758	0.808

*CFA* confirmatory factor analysis, *RMSEA* root mean square error of approximation, *CI* confidence interval, *TLI* Tucker-Lewis index, *CFI* comparative fit index

We decided to conduct a post-hoc analysis in an attempt to improve the fit of the model. The post-hoc modification (Model 2) concerned the four reverse-coded items; three items in the Mutual Support dimension and one item in the Communication dimension. Goodness of fit statistics related to Model 2 showed that the correlations between the error variances for the four items made a moderately improvement to model fit. The normed chi-square decreased from 3.43 to 2.96, RMSEA decreased from 0.076 to 0.068, while TLI and CFI increased slightly (see Table 3).

**Fig. 1:**
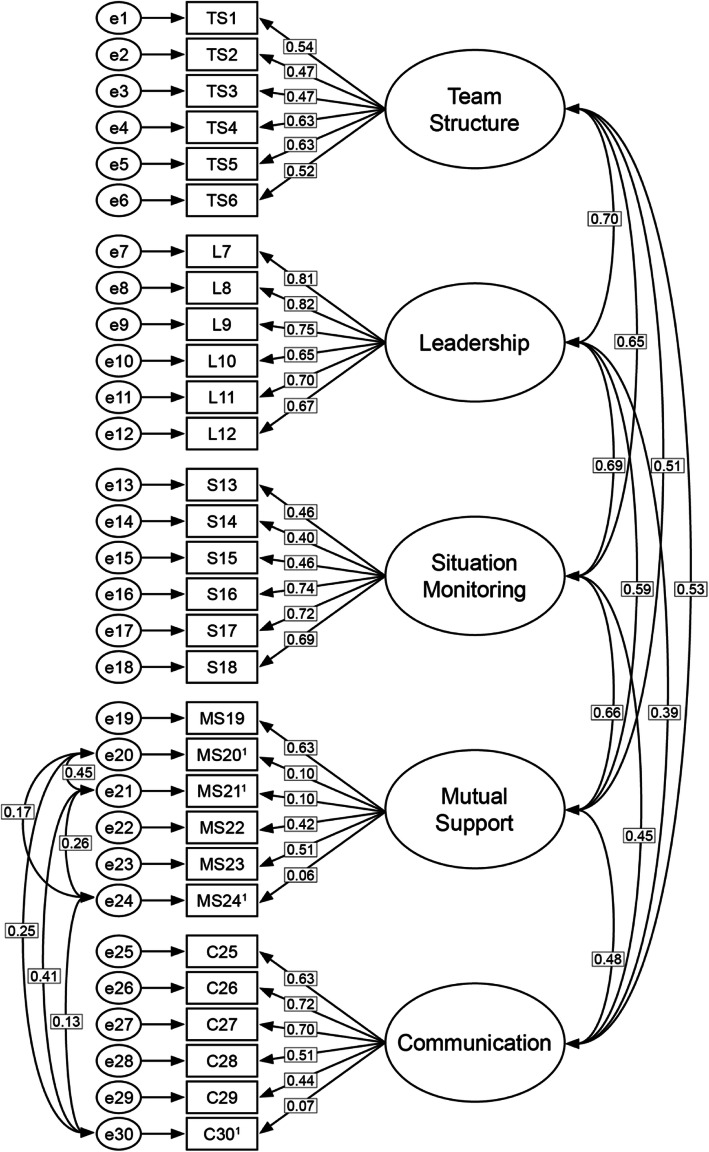
Confirmatory analysis model (Model 2) with factor loadings and correlations.^1^ = reverse-coded items

Figure 1 presents factor loadings and correlations between the factors (dimensions) and between the four reverse-coded items. The factor loadings ranged from 0.06 to 0.82 and the correlations between the dimensions were between 0.39 and 0.70. The correlations between the four reverse-coded items ranged between 0.13 and 0.45.

## Discussion

This study aimed to test the reliability and structural validity of the Swedish version of the T-TAQ, and the results revealed that the questionnaire has some potential to measure healthcare professionals’ attitudes toward teamwork. Cronbach’s alpha showed that the reliability of three of the dimensions (Team Structure, Leadership, Situation Monitoring) and the total T-TAQ were considered acceptable, with values above 0.70 [28]. In contrast, the Cronbach’s alpha values were notably lower in the two dimensions with negatively worded items (Mutual Support, Communication). The validation study of the original English version of the T-TAQ showed acceptable values in all dimensions (Team Structure 0.70, Leadership 0.81, Situational Monitoring 0.81, Mutual Support 0.70, Communication 0.74) [20]. In the Norwegian study by Ballangrud et al. [32], there were low values in the same two dimensions as in our study but also in the Team Structure dimension. In the study by Sweigart et al. [9], the lowest Cronbach’s alpha value was observed in the Communication dimension (0.57). Polit and Yang [28] note that the internal consistency of a questionnaire is not a property of the questionnaire itself but rather a property of the questionnaire used with a particular population under specific circumstances. Moreover, the estimates of internal consistency can be affected by whether a measure captures both the main construct and other personal attributes, such as social desirability responses leading to bias [28].

The results of the corrected item-total correlation and the factor loadings in the CFA indicate that there may be a problem with the negatively worded items in the Mutual Support and Communication dimensions. In general, negatively worded items should be avoided, as including both negative and positive items on a scale can confuse people by reversing polarities [28]. However, we recommend not removing these items as a multicenter study is required that validates the questionnaire.

A post-hoc modification (Model 2) with a correlation between the reverse-coded items resulted in a model with better fit to the data. The RMSEA value was close to 0.06 (0.068) and the normed Chi-square was below 3. According to the recommendations for CFA, these goodness-of-fit indexes of CFA indicated an acceptable fit with the original construct [30]. Compared to the study by Ballangrud et al. [32], who validated the Norwegian version of the T-TAQ in healthcare professionals, the values reported herein were slightly higher. On the other hand, the TLI and CFI were below the recommended goodness-of-fit indexes (> 0.95), but were higher than in the previous validation study [32]. The correlations, conducted as a part of CFA, between the five dimensions suggest that all dimensions overlap to some degree but also demonstrate unique variance. Post-hoc modification can improve the fit, but it is important that the final model does not differ from the theoretical model [33].

The sample size is an important factor in CFA, and it is desirable to have a sample size of at least 10 cases for each item [33]. Although the present study had 423 cases in the CFA, even more are preferred [28].

In addition to the study by Ballangrud et al. [32], this is the second study to use CFA to investigate the T-TAQ, which was not used in the original T-TAQ validation study by Baker et al. [20]. Although the T-TAQ has been used to evaluate team training in several studies, we have not found any study where CFA has been performed. Baker et al. [20] recommended additional validation with CFA since the T-TAQ is based on a theoretical framework of teamwork.

### Limitations

The response rate was low, and therefore, there is a risk of sampling bias, which limits the generalizability of the study [34]. The low response rate may have been due to high workloads and stress. In addition, the motivation to respond to the questionnaire may be affected by whether the concept of teamwork has been acknowledged or whether an item is relevant to the responder. It was not possible to carry out a dropout analysis of those who did not respond since the healthcare professionals answered the questionnaire anonymously. Within the various profession groups, the proportion of physicians and allied health professionals who answered the questionnaire was lower compared to midwives, registered nurses and assistant nurses. However, none of the profession groups was underrepresented in the sample.

## Conclusions

This validation study of the Swedish version of the T-TAQ indicates that the questionnaire has some potential for measuring healthcare professionals’ individual attitudes toward the core components of teamwork. Continued use in both research and education should take into account that the T-TAQ can be used as unidimensional, and responders should pay attention to negatively worded items. Further validation of the Swedish version of the T-TAQ is required. It would be desirable to conduct studies with both healthcare professionals from various healthcare settings and students from various disciplines and educational levels.

## Data Availability

The datasets used and analyzed during the current study are available from the corresponding author on reasonable request.

## Abbreviations

AHRQ: Agency for Healthcare Research and Quality
CFA: Confirmatory factor analysis
CFI: Comparative fit indexes
CI: Confidence interval
RMSEA: Root mean square error of approximation
T-TAQ: TeamSTEPPS Teamwork Attitudes Questionnaire
TeamSTEPPS: Team Strategies and Tools to Enhance Performance and Patient Safety
TLI: Tucker-Lewis index
WHO: World Health Organization
